# Gastrointestinal failure score in children with traumatic brain injury

**DOI:** 10.1186/s12887-021-02673-5

**Published:** 2021-05-04

**Authors:** Ying Zhou, Weifeng Lu, Weibing Tang

**Affiliations:** Department of Pediatric Surgery, Children’s Hospital of Nanjing Medical University, 72 Guangzhou Road, Jiangsu Province 210008 Nanjing, China

**Keywords:** Gastrointestinal failure (GIF) score, Traumatic brain injury, Children

## Abstract

**Background:**

To review the value of the gastrointestinal failure (GIF) score in children with different degrees of traumatic brain injury (TBI) by analyzing the correlation between outcome and gastrointestinal function.

**Methods:**

A total of 165 children with TBI who were diagnosed and treated in the surgical intensive care unit (SICU) for longer than 72 h between August 2017 and September 2019 were analyzed. Admission parameters included sex, age, Glasgow Coma Scale (GCS) score, body mass index (BMI), leukocyte count, C-reactive protein (CRP), hemoglobin (Hb), hematocrit (Hct), blood glucose, lactic acid, procalcitonin (PCT), albumin, plasma osmotic pressure, prothrombin time (PT) and activated partial thromboplastin time (APTT). To predict outcomes, the Pediatric Sequential Organ Failure Assessment (SOFA) score, Pediatric Clinical Illness Score (PCIS), and mean GIF score for the first three days were combined.

**Results:**

The percentage of patients with gastrointestinal dysfunction on the first day was 78.8 %. Food intolerance (FI) and intra-abdominal hypertension (IAH) developed in 36.4 and 21.8 % of the patients, respectively. The GIF score and mean GIF score for the first three days were significantly different between children with different degrees of TBI (*P* < 0.05); these scores were also significantly different between patients who died and those who survived (*P *< 0.05). The mean GIF score for the first three days was identified as an independent risk factor for mortality (odds ratio > 1, 95 % confidence interval = 1.457 to 16.016, *P* < 0.01), as was the PCIS. Receiver operating characteristic (ROC) curve analysis suggested that the mean GIF score for the first three days had the same calibrating power as the PCIS in discriminating the risk of death of children.

**Conclusions:**

The incidence of gastrointestinal dysfunction in children with TBI is high. The GIF score has the ability to reflect the status of the gastrointestinal system. The mean GIF score for the first three days has high prognostic value for ICU mortality in the SICU.

## Background

Traumatic brain injury (TBI) has the highest mortality and morbidity of all types of trauma and represents a serious threat to the life and physical health of children. According to statistics, the incidence of TBI in European and American countries is as high as 150–200/100,000/year, while the incidence is approximately 100–150/100,000/year in China [1, 2]. Due to their young age, low crisis awareness and poor self-protection ability, severe TBIs resulting in a Glasgow Coma Scale (GCS) score of less than 8 are very common in children. Children with such TBIs have a mortality rate of 20 % and a severe disability rate of > 50 % [3] and require monitoring and treatment in intensive care units (ICUs). Children in ICUs with gastrointestinal bleeding, dysfunction or failure often endure prolonged hospital stays and have increased mortality [4]. However, objectively evaluating gastrointestinal dysfunction is difficult because quantitative standards to classify severity are lacking [5, 6]. Few of the various existing scoring systems for determining the severity of disease in pediatric patients include an evaluation of gastrointestinal function [7]. The gastrointestinal failure (GIF) score serves as an objective indicator used to evaluate gastrointestinal dysfunction [8]. This study collected the clinical data of children with TBI admitted to the surgical intensive care unit (SICU) in our hospital, and GIF scores were estimated. The purpose of this study was to reveal the importance of gastrointestinal dysfunction and its impact on the prognosis of children with TBI and to provide reliable evidence for the evaluation of gastrointestinal function in children with TBI.

## Methods

### Ethics

Written informed consent was obtained from the patients and/or their parents, and this project was approved by the ethics committee of Children’s Hospital of Nanjing Medical University (No. 202001004-1).

### Clinical information

We conducted a prospective observational study at Children’s Hospital of Nanjing Medical University in China. We included patients admitted from August 2017 to Sept 2019 who met the following criteria: (a) age 3 months to 13 years and 8 months; (b) admission with TBI (defined as severe with GCS score from 3 to 8, moderate with GCS score from 9 to 12 and mild with GCS score from 13 to 15) within 24 h of injury; (c) confirmation of intracranial injury on head CT as brain edema, subdural hemorrhage and intracranial hemorrhage; (d) no treatment with sedatives; and (e) no history of previous intracranial or gastrointestinal disease. We excluded patients with (a) neonatal age (< 28 days); (b) TBI combined with primary gastrointestinal injury; (c) admission to the hospital more than 24 h after injury; (d) hospital stay shorter than 72 h; and (e) TBI combined with organ failure, serious metabolic disorders, and other basic diseases.

The clinical parameters that were collected on admission included sex, age, body mass index (BMI), GCS score, leukocyte count, C-reactive protein (CRP) level, hemoglobin (Hb), hematocrit (Hct), blood glucose (Glu), lactic acid (Lac), procalcitonin (PCT), albumin (ALB), plasma osmotic pressure (POP), prothrombin time (PT) and activated partial thromboplastin time (APTT). The Sequential Organ Failure Assessment (SOFA) score and Pediatric Clinical Illness Score (PCIS) were recorded daily. The gastrointestinal function of the patients was evaluated daily by the GIF score as follows [8]: 0 = normal gastrointestinal function; 1 = enteral feeding with under 50 % of the calculated need; 2 = food intolerance (FI) or intra-abdominal hypertension (IAH); 3 = FI and IAH; and 4 = abdominal compartment syndrome (ACS). The SOFA scores, PCIS and mean GIF scores for the first three days were combined to predict outcome. The primary outcome parameter was ICU mortality.

A few caveats should be noted. Enteral feeding was provided as early as possible. Stress ulcers were diagnosed if dark blood fluid was found in gastrointestinal decompression tubes or gastric and duodenal mucosa erosion and ulcers were observed by gastroscopy. The criteria for FI diagnosis were failed enteral feeding or vomiting after eating more than three times a day, gastric residual volumes exceeding 50 % of the feeding volume, and intestinal obstruction, severe diarrhea, or bloating that could not be resolved within 24 h. If FI developed, intra-abdominal pressure (IAP) was measured with an empty bladder in the supine position using the closed-loop system repeated-measurements technique [9]. IAP was measured at least twice per day when normal. When the IAP was higher than 12 mmHg, it was measured four times per day at different points. IAH was defined as a persistent IAP of 12 mmHg or higher. ACS was defined as a persistent IAP of 20 mmHg or greater accompanied by new organ failure. The mean and maximum IAP values were documented daily, and the mean value was used to calculate the daily GIF score.

### Statistical analysis

SPSS 19.0 (Professional Edition) was used for data analysis. The data are presented as the mean ± standard (`*x* ± s) unless stated otherwise. Differences between two groups were evaluated by the two-sample T test for continuous variables and by the chi-square test (or Fisher’s exact probability) for categorical variables. One-way ANOVA was used to compare multiple means. GIF scores for the first three days were calculated as the mean individual score for three days for every child. Risk factors for ICU mortality were identified by univariate analyses of admission parameters, and parameters with *p* < 0.1 were entered into a multiple logistic regression model to identify independent risk factors. The collinearity of the parameters in the regression was checked. Receiver operating characteristic (ROC) curves were used to determine the likelihood ratios for the abilities of the GIF score, SOFA score and PCIS to predict ICU mortality. *P* < 0.05 was considered statistically significant.

## Results

This study included 165 children (103 boys (62.4 %) and 62 girls (37.6 %)) with an average age of 4 years and 11 months. There were 92 cases of severe, 23 cases of moderate and 50 cases of mild craniocerebral injury; the incidence rate of stress ulcers was much higher in the severe group than in the moderate and mild groups (85.9 %, 4.3 %, and 0 %, respectively, *p* < 0.05), as was the incidence rate of secondary gastrointestinal dysfunction (100 %, 82.6 %, and 38 %, respectively). The GIF score on the first day and the mean GIF score for the first three days differed significantly among the three groups (*p* < 0.05, Table 1).

**Table 1:** Admission and outcome parameters for different degrees

Parameters	Severe	Moderate	Mild	p
Number (%)	92(55.8)	23(13.9)	50(30.3)	
Age	4.70 ± 3.57	5.08 ± 4.63	5.05 ± 4.03	F = 0.18, *p* = 0.84
BMI (kg/m^2^)	16.46 ± 2.99	18.08 ± 5.06	16.81 ± 2.96	F = 2.17, *p* = 0.12
Stress ulcer (%)	85.90	4.30	0.00	*X*^*2*^ = 116.48^2^, *p* < 0.05
GSC	4.96 ± 1.82	10.83 ± 1.07	13.50 ± 0.51	F = 606.14, *p* < 0.05
Incubation (%)	87(94.57)	2(8.70)	0(0)	*X*^*2*^ = 138.62^2^, *p* < 0.05
SOFA score	7.86 ± 2.77	4.00 ± 1.81	2.10 ± 1.18	F = 110.03, *p* < 0.05
PCIS score	73.91 ± 10.36	91.48 ± 7.70	95.80 ± 3.66	F = 120.43, *p* < 0.05
First day GIF score	2.28 ± 0.56	0.96 ± 0.56	0.60 ± 0.95	F = 104.16, *p* < 0.05
Mean GIF score for the first three days	2.02 ± 0.44	0.57 ± 0.38	0.42 ± 0.75	F = 165.62, *p* < 0.05
Mortality (%)	43.5	0	0	*X*^*2*^ = 41.90^2^, *p* < 0.05

Unit of measure provided in parentheses

A total of 130 patients (78.8 %) had gastrointestinal dysfunction on the first day of hospital admission, including 34 with insufficient feeding (20.6 %), 60 with FI (36.4 %), and 36 with IAH or ACS (21.8 %). Eighty children had TBI with stress ulcers (48.5 %). The children were divided into two groups based on whether FI occurred on the first day of admission. Sixty-nine children (41.8 %) had a GIF score < 2 on the first day, and 96 children (58.2 %) had a GIF score ≥ 2. Significant differences were observed in GCS and GIF scores on the first day, the mean GIF scores for the first three days of admission, SOFA scores, and PCISs between the two groups (*p* < 0.05, Table 2).

**Table 2:** Admission and outcome parameters for GIF score < 2 and GIF score ≥ 2

Parameters	GIF score < 2	GIF score ≥ 2	p
Number(%)	69(41.8)	96(58.2)	
Age	4.82 ± 4.23	4.96 ± 3.64	t = 0.23, p = 0.82
BMI (kg/m^2^)	17.11 ± 3.37	16.56 ± 3.36	t=-1.03, p = 0.30
GCS	12.29 ± 2.00	5.54 ± 2.83	t = 17.91, *p* < 0.05
First day GIF score	0.49 ± 0.50	2.38 ± 0.49	t=-24.02, *p* < 0.05
Mean GIF score for the first three days	0.31 ± 0.36	2.07 ± 0.38	t=-29.86, *p* < 0.05
SOFA score	2.87 ± 1.80	7.52 ± 3.06	t=-12.23, *p* < 0.05
PCIS score	94.09 ± 6.17	75.02 ± 11.24	t = 13.96, *p* < 0.05
Mortality (%)	0	41.67	*X*^*2*^ = 37.95^2^, *p* < 0.05

Unit of measure provided in parentheses

### Multivariate regression and receiver operating characteristic (ROC) curve analyses

**Fig. 1:**
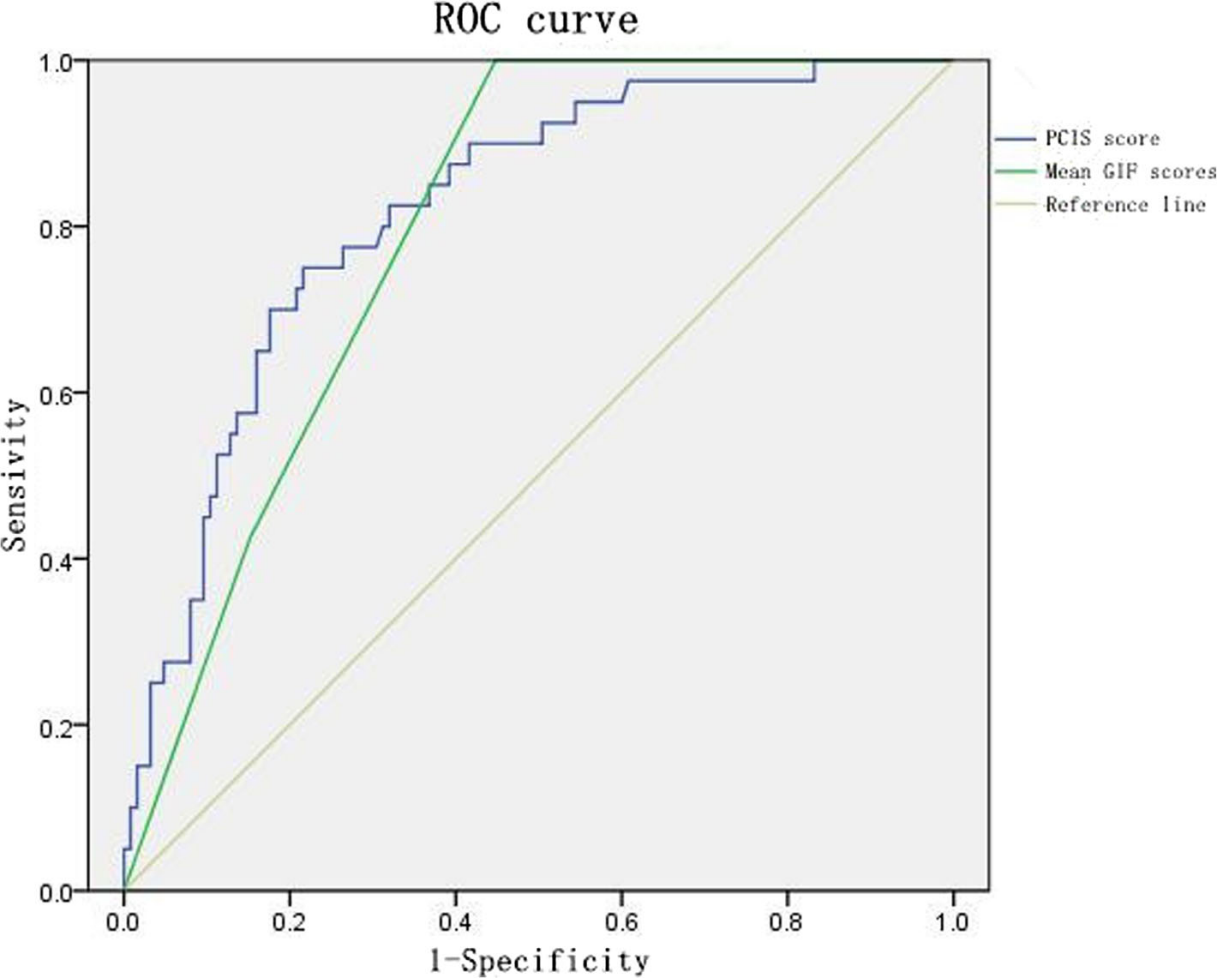
ROC curves for the PCIS and mean GIF score for the first three days

Lab parameters except C-reactive protein, hematocrit, procalcitonin and plasma osmotic pressure were significantly different between the deceased and survival groups (*p* < 0.05). There was no collinearity among the parameters in the regression (VIF < 5). Binary multivariate logistic regression analysis was performed using the SOFA score, PCIS, GIF score on the first day of admission, and mean GIF score for the first three days to establish a risk of death prediction model. The overall accuracy rate of this model in predicting death was 90.9 %. The PCIS and mean GIF score for the first three days were independent risk factors for death (odds ratio (OR) > 1, Table 3). ROC analysis showed that the PCIS and the mean GIF score for the first three days had good predictive ability for the death of children with TBI (Fig. 1).

**Table 3:** Logistic regression analysis for different scores

	B	S.E,	Wals	df	Sig.	Exp (B)	95% C.I	
							Lower limit	upper limit
SOFA score	-0.14	0.17	0.70	1	0.40	0.87	0.63	1.20
PCIS	0.25	0.06	15.64	1	0.00	1.29	1.14	1.46
First day GIF score	-0.66	0.68	0.94	1	0.33	0.51	0.14	1.97
Mean GIF score for the first three days	1.58	0.61	6.63	1	0.01	4.83	1.46	16.02
constant	-17.96	5.87	9.37	1	0.00	0.00		

## Discussion

In this study, 135 children with TBI (78.8 %) had gastrointestinal dysfunction on the first day of admission, suggesting that gastrointestinal dysfunction in children with TBI is very common. The incidence of stress ulcers in children with TBI was 48.5 % and gradually increased with TBI severity, with a rate of 85.9 % in severe cases, suggesting that the risk of gastrointestinal mucosal ischemia is high, which is consistent with previous reports in adult TBI patients [10]. The gastrointestinal tract is the only system that is jointly controlled by the central nervous system, enteric nerves, and autonomic nerves; thus, the complex neuroendocrine network named the brain-gut axis plays an important role in regulating gastrointestinal function. After the central nervous system is damaged, various pathways in the brain-gut axis are blocked, and many brain-gut peptides are secreted abnormally. These pathways and peptides cannot transmit information normally or stimulate gastrointestinal motility [11–13]. The gastrointestinal mucosa is in a state of hypoperfusion, resulting in the spread of focal small ulcers, which usually manifest as stress ulcers. In addition, gastrointestinal hormone levels and secretion are disordered, and intestinal flora are imbalanced. Feedback from the gastrointestinal tract to the central nervous system is abnormal, causing gastrointestinal dysfunction [14–16].

Approximately one third of the children developed FI on the first day of ICU admission. These children were significantly more ill (lower GCS scores and higher SOFA scores and PCISs) and exhibited greater morality than those without FI. The prevalence of FI was lower than that in previous studies of adult critically ill patients [17, 18]. The reasons for this discrepancy are not clear, but we speculate that the following factors may be involved. (1) The included children did not have primary gastrointestinal injury. Compared with elderly critically ill patients with cardiovascular disease or diabetes, the children’s organ function was basically normal. (2) Children’s nervous systems are still developing; therefore, the stress response may not be completely elicited, and the abnormal release of brain-gut peptides is weakened [19]. (3) The gastrointestinal system of children can adapt to changes in the structure and function of neuronal circuits, and mucosal repair and functional reconstruction mechanisms are stronger in children than in adults [20]. Although we defined FI using objective measurements for greater precision, FI is a subjective variable and a universally used clinical characteristic covering the entire spectrum of gastrointestinal symptoms. FI allows a functional assessment with some clinical relevance [21], as shown in our study. IAH did not occur as frequently as FI in our study, and ACS occurred less often. References to adult evaluation criteria are of somewhat limited value. Further studies are needed to standardize the evaluation criteria.

The incidence of secondary gastrointestinal dysfunction increased with injury severity, and the GIF score on the first day and the mean GIF score for the first three days differed significantly among the severe, moderate and mild groups. The intestinal mucosa morphology was found to change within a short time after trauma, including epithelial cell detachment and apoptosis, rupture of the villi, edema of interstitial tissue and the lamina propria, interruption of tight junctions, etc., and the mucosal barrier was observed to have lost its protective function [11]. Additionally, the intestinal flora was completely disordered within a few hours after injury, and the microbial composition and relative abundance changed significantly. The number of beneficial microbiota decreased, while pathogenic flora, which showed relatively increased invasiveness and virulence, dominated the intestinal tract, and the diversity and stability of the microbial ecological system were destroyed. More severe trauma corresponded to worse dysbiosis and a greater effect on gastrointestinal function, leading to a high GIF score. The infection risk and the mortality rate of these pediatric patients were substantially increased [12].

The comparison of the relevant clinical indicators in the deceased and survival groups indicated that the GIF score on the first day and the mean GIF score for the first three days were significantly higher in the deceased group than in the survival group, as were the SOFA scores and PCISs. Multivariate logistic regression analysis suggested that a high mean GIF score for the first three days was closely related to mortality as an independent risk factor. The GIF score reflects gastrointestinal function, which can be classified into different levels, similar to other scoring systems for organ function failure. The clinical value and high reliability of the GIF score for predicting outcomes have been verified in intensive care patients and in digestive system diseases [10, 16, 22]. Although the GIF score can be used as an independent risk factor for predicting the risk of death in critically ill patients [23], it focuses on gastrointestinal function at the time of injury. However, the condition of a child develops and changes during hospitalization. The reliability of the GIF score on the first day was relatively low, and its role in predicting death during the entire ICU stay is limited [24]. The mean GIF score for the first three days can be used to dynamically observe and assess changes in gastrointestinal dysfunction during peak disease development, providing better continuity. Reintam et al. [8] found that the mean GIF score for the first three days was more important in predicting death than the GIF score on the first day. In this study, the mean GIF score for the first three days but not the GIF score on the first day was considered an independent risk factor. The reliability of the latter score was relatively low, suggesting that although the GIF score can be used as an objective indicator, the effectiveness and accuracy of dynamic observation and scoring are even higher. The mean GIF score for the first three days was better than the GIF score on the first day for evaluating the gastrointestinal function of children with TBI. One limitation of this study is that the SOFA score may be inapplicable to young infants and toddlers in terms of the items assessed [25–27]. The PCIS fully integrates the physiological and morbidity characteristics of children at different ages and was introduced to predict the risk of death [28, 29]. In this study, the mean GIF score for the first three days had a predictive ability for death comparable to that of the PCIS. Both had good predictive abilities for the risk of death, again confirming the clinical significance of the GIF score in diagnosing gastrointestinal dysfunction in children with TBI and further emphasizing the importance of continuous monitoring and dynamic observation of the gastrointestinal status of children at different time points. Organ dysfunction in critically ill patients should be scored dynamically [30].

## Conclusions

In summary, the incidence rate of gastrointestinal dysfunction in children with TBI is high. The GIF score can accurately classify and objectively assess gastrointestinal status. A high GIF score is significantly correlated with ICU mortality. As an independent risk factor, the mean GIF score for the first three days has higher value for predicting ICU mortality than the GIF score on the first day; this result can provide guidance for the clinical evaluation and treatment of gastrointestinal dysfunction in children with TBI.

## Data Availability

All data can be found in the medical records system of Children’s Hospital of Nanjing Medical University. Since each piece of data corresponds to an identified patient, the details are inconvenient to disclose.

## Abbreviations

TBI: Traumatic brain injury
SICU: Surgical intensive care unit
GCS: Glasgow Coma Scale
BMI: Body mass index
CRP: C-reactive protein
Hb: Hemoglobin
Hct: Hematocrit
PCT: Procalcitonin
PT: Prothrombin time
APTT: Activated partial thromboplastin time
SOFA: Sequential Organ Failure Assessment
PCIS: Pediatric Clinical Illness Score
GIF: Gastrointestinal failure
FI: Food intolerance
IAH: Intra-abdominal hypertension
ACS: Abdominal compartment syndrome
IAP: Intra-abdominal pressure
ROC: Receiver operating characteristic
